# Current attitudes toward drug checking services and a comparison of expected with actual drugs present in street drug samples collected from opioid users

**DOI:** 10.1186/s12954-023-00821-x

**Published:** 2023-07-07

**Authors:** James A. Swartz, Marya Lieberman, A. David Jimenez, Mary Ellen Mackesy-Amiti, Heather D. Whitehead, Kathleen L. Hayes, Lisa Taylor, Elizabeth Prete

**Affiliations:** 1 Jane Addams College of Social Work, University of Illinois Chicago, 1040 W. Harrison Street, (MC 309), Chicago, IL 60607 USA; 2 Department of Chemistry and Biochemistry, University of Notre Dame, Notre Dame, USA; 3 Community Health Sciences, School of Public Health, University of Illinois Chicago, Chicago, USA; 4 Chestnut Health Systems, Chicago, IL USA; 5 Thresholds Homeless Outreach Program, Chicago, IL USA

**Keywords:** Drug checking services, Harm reduction, Fentanyl, Opioid overdose-related fatalities, Syringe program services, Substance use

## Abstract

**Background:**

The opioid epidemic continues to be associated with high numbers of fatalities in the USA and other countries, driven mainly by the inclusion of potent synthetic opioids in street drugs. Drug checking by means of various technologies is being increasingly implemented as a harm reduction strategy to inform users about constituent drugs in their street samples. We assessed how valued drug checking services (DCS) would be for opioid street drug users given the ubiquity of fentanyl and related analogs in the drug supply, the information they would most value from drug checking, and compared expected versus actual constituent drugs in collected samples.

**Methods:**

A convenience sample of opioid street drug users (*N* = 118) was recruited from two syringe service exchange programs in Chicago between 2021 and 2022. We administered brief surveys asking about overdose history, whether fentanyl was their preferred opioid, and interest in DCS. We also collected drug samples and asked participants what drug(s) they expected were in the sample. Provided samples were analyzed using LC–MS technology and the results compared to their expected drugs.

**Results:**

Participants reported an average of 4.4 lifetime overdoses (SD = 4.8, range = 0–20) and 1.1 (SD = 1.8, range = 0–10) past-year overdoses. A majority (92.1%) believed they had recently used drugs containing fentanyl whether intentionally or unintentionally. Opinions about the desirability of fentanyl were mixed with 56.1% indicating they did not and 38.0% indicating they did prefer fentanyl over other opioids, mainly heroin. Attitudes toward DCS indicated a general but not uniform receptiveness with a majority indicating interest in DCS though sizeable minorities believed DCS was “too much trouble” (25.2%) or there was “no point” in testing (35.4%). Participants were especially inaccurate identifying common cutting agents and potentiating drugs such as diphenhydramine in their samples (sensitivity = .17).

**Conclusions:**

Results affirmed street drug users remain interested in using DCS to monitor their drugs and such services should be more widely available. Advanced checking technologies that provide information on the relative quantities and the different drugs present in a given sample available at point-of-care, would be most valuable but remain challenging to implement.

## Background

The decades-long opioid epidemic continues to manifest in persistently high rates of opioid-related overdoses and fatalities. After a brief decline in 2018, the US national opioid-related overdoses and fatalities rate increased again, owing to the expanded use of potent synthetic opioids, primarily illicitly manufactured fentanyl, to augment or completely supplant heroin as well as to the potentiating effects of the SARS-CoV-2 pandemic [1–4]. Recent seizure-based and testing data indicate fentanyl is now incorporated into non-opioid drugs such as cocaine and methamphetamine or marketed as a different drug entirely such as alprazolam (i.e., Xanax®), hydrocodone (i.e., Vicodin®) or oxycodone (i.e., Oxycontin®) in illegally manufactured pills [1, 5–7]. Between 2020 and 2021, US opioid-related overdoses and fatalities increased 15.4% from 70,029 to 80,816, 71,239 (88.3%) of which were attributable to fentanyl and related synthetic opioids [8].

The public health response to reduce opioid-related overdoses and fatalities and stem the epidemic has been multifold and has included efforts to: increase access to medications for opioid use disorder (MOUDs) such as methadone and buprenorphine (e.g., Suboxone®); increase use of non-opioid-based treatment for pain; reduce use of prescription opioids through state-implemented prescription drug monitoring programs (PDMP); and broaden the provision of overdose education and naloxone distribution (OEND) programs [9–12]. Recently, multiple strategies collectively termed “drug checking services” (DCS) have emerged and are gradually being adopted as an additional means of harm reduction to decrease opioid-related overdoses and fatalities.

Drug checking was first used in the USA in the late 1960s to test illegal drugs that had become prevalent at the time such as LSD [13]. Subsequently, DCS was most widely adopted in Europe as a means for testing “party drugs” sold in nightclubs and similar venues [14]. It has only been over the past 5–7 years in response to the opioid epidemic, that DCS has expanded as a harm reduction strategy in North America; first implemented in Canada and subsequently in larger US cities such Baltimore, Boston, Chicago, Providence, and San Francisco [15, 16]. An emergent use of DCS is to monitor street drug markets, which by virtue of being unregulated and decentralized, produce drugs that vary widely in terms of the concentrations of fentanyl, other drugs such as benzodiazepines commonly used to potentiate the effects of street drugs sold as opiates, and cutting agents that are not always benign with respect to their health effects [17, 18]. The unpredictable variability of the illegal drug market has itself become an overdose risk factor [19–21].

DCS can be implemented in multiple settings using multiple technologies, each with pros and cons [22–25]. Settings include providing users with the means to test their own drugs often in conjunction with other harm reduction services at venues such as syringe service programs (SSPs), supervised injection sites (SIS), or via sample mail-in [26]. Checking technologies vary as well, ranging from the inexpensive and simple-to-use fentanyl or benzodiazepine test strips to complex and expensive but highly sensitive and accurate lab-based equipment requiring trained technicians such as gas chromatography mass spectrometry (GC–MS) [25, 26].

Early research ahead of widespread distribution of fentanyl test strips, which have been the primary way DCS has been implemented in the USA, focused on whether users were willing to adopt drug checking prior to using newly purchased street drugs [15, 27–29]. These studies generally found street drug users considered DCS as beneficial and indicated intent to use DCS to check their drugs prior to use. Later research on the effectiveness of DCS for reducing overdose risk behaviors has found DCS influences both intent to use and overdose risk reduction behaviors [13, 28].

Despite these promising early findings, barriers to adoption remain such as users over trusting their drug sellers, difficulties accessing DCS, aggressive policing policies and related legal concerns, and the expense of training and equipment for the more technologically advanced drug checking technologies [13, 30]. Even barriers to adoption of the simplest means of drug checking, fentanyl test strips, remain and include availability of supplies and confusion over how to interpret the results [31]. Absent widely available DCS, or simply remaining reluctant to use DCS, some users adopt less reliable and less valid means of determining whether fentanyl is present in their street drugs, for example believing they can discern the presence of fentanyl simply by the look or “taste” of the drugs [32].

Continued research on DCS is now focusing on how drug checking is best implemented to determine the most effective way to present and use DCS to prevent overdoses [30]. Given that drug markets in North America have changed just over the past 5 years as DCS was being implemented more broadly, with fentanyl now expected to frequently supplement or supplant heroin [19], one could reasonably ask if street drug users still believe non-quantitative (i.e., indicating presence or absence but not amount) DCS methods such as fentanyl test strips continue to provide useful information. Although detecting the presence or absence of fentanyl remains clearly informative when checking for fentanyl in non-opioid street drugs sold as cocaine, alprazolam, or MDMA where the presence of fentanyl is not expected or less likely to be expected [7, 33–35], there is a question as to whether detecting the presence or absence of fentanyl still provides useful information for opioid street drug users for whom the presence of fentanyl is expected if not sought after [36, 37].

It is also possible that drug checking methods such as infrared absorption spectroscopy, which can provide both quantitative and qualitative information on fentanyl and other (unexpected) drugs that could be mixed into a street sample but which is less sensitive to fentanyl than the test strips and requires a trained technician, could provide more useful information if it were more widely available [25]. However, recent qualitative research has yielded mixed results with some users indicating fentanyl test strips are useful while another study reports low enthusiasm for DCS generally given structural barriers such as having to give up part of their drugs for testing, accessibility, and limited recourse following a positive fentanyl test result [37].

In the current study, we surveyed street drug users who primarily use any opioid such as heroin, morphine, fentanyl and related analogs, or illegally manufactured or purchased oxycodone, hydrocodone, or codeine, to assess their interest in using DCS if these were to become more available locally. We specifically wanted to assess if fentanyl detection is still perceived as useful and worth the time and effort, as well as how they intended to use drug testing results and what information they would most value from DCS. We then compared what specific drugs users expected compared with what drugs were in their provided samples as determined by LC–MS testing. We also examined whether having experienced an overdose is positively associated with an increased intent to use DCS as has been demonstrated in prior research [38].

## Methods

### Study setting

Data were collected as part of project to assess a novel method for testing street drugs, the illegal drug Paper Analytic Device (idPAD). The idPAD is a paper strip that has been chemically treated and can detect a number of different drugs in a single use. It has been successfully used to detect substandard or counterfeit prescription drugs sold in low resource countries. The parent study for this project assessed whether the idPAD can be adapted to accurately test illegally manufactured drugs sold on the street as heroin or other opioids. Detailed information on use of the idPAD in this study as well as the design and development of the idPAD is available elsewhere [39, 40].

Between August 2021 and April 2022, we recruited a convenience sample of participants from an SSP operating at two community-based outreach offices on Chicago’s northwest and west sides. The two sites are in socioeconomically disadvantaged communities composed disproportionately of racial/ethnic minorities, and have relatively high rates of opioid-related overdoses and fatalities among all Chicago community areas [41]. The study protocol was reviewed and approved by the UIC and Notre Dame institutional review boards.

Recruitment was done through posted fliers at both locations and through program staff word-of-mouth. Study inclusion criteria were: (1) at least 18 years of age; (2) past-month opioid or cocaine user; (3) speaking and understanding at least conversational English; (4) a client of the syringe service exchange program where recruitment occurred; and (5) willing to provide a same-day sample of a recently purchased street drug for testing.

In total, the study recruited 138 participants of which 123 (83.1%) provided both a drug sample and interview with the remaining 15 (16.9%) providing only a drug sample. Five of the samples could not be tested using LC–MS owing to contamination or too small an amount for valid testing was provided, reducing the analytic N with full information to 118.

### Procedures

Study recruits were first read a description of the study and what their involvement would entail. If they agreed to participate, a research assistant (RA) obtained verbal consent. Study participation consisted of first swiping a small amount of their drug across the face of an unused idPAD and completing a 15-to-20-min survey administered via REDCap [42] by the RA. Participants were paid $15 for providing the drug sample and an additional $10 if they agreed to the survey.

All surveyed participants provided a sample of their recently purchased street drug (10–15 mg of a powder or 24 uL of a liquid drug) by swiping it across the face of an unused idPAD. An RA then dipped the idPAD into a small water tray, so the hydrophilic wax coating on the device could draw water across the card, dissolving the drug sample [40]. The collected drug samples were dried and express mailed in weekly batches as they were collected to the chemistry lab at Notre Dame for LC–MS analysis.

Samples were tested for thirteen different drugs or drug classes (and additional metabolites) participants were asked about on the survey with the exceptions of codeine and buprenorphine. The drugs tested included: morphine, heroin, fentanyl, fentanyl analogs, hydromorphone, hydrocodone, tramadol, cocaine, methamphetamine, diphenhydramine, lactose, mannitol, and quinine. Samples were also tested for three drugs not asked about: oxymorphone, phencyclidine (PCP), and levamisole, a cutting agent most often found in powder and crack cocaine and which can have serious health effects such as agranulocytosis [17].

Drugs that were present in amounts that could be quantified and not simply present as trace amounts were coded as present in the sample. We defined trace amounts as quantities above the LC–MS limit of detection (LOD) but below the limit of quantification (LOQ) [43]. These concentrations are all below 1 ng/mL, which means the maximum amount of the trace components in the original drug sample was well below 0.001%, (i.e., less than 1 µg in a 10 mg drug dose) and not present in large enough concentrations to have an effect on the user [43]. LC–MS results indicated as present/absent were sent to UIC where they were combined with the survey data in REDCap for analysis.

### Measures

The main independent variable was self-reported number of opioid-related lifetime overdoses, capped at 20 or more. After reviewing the response distribution, we divided participants into 3 groups: 0–1 overdoses (*n* = 33, 28.0%); 2–5 overdoses (*n* = 54, 46.8%); and 6+ overdoses (*n* = 31, 26.3%). We disaggregated the remaining study measures by these 3 groups to determine whether their overdose histories affected attitudes toward DCS and what drugs participants expected were in the provided sample. Other overdose-related information included: number of past-year overdoses; their most recent overdose measured categorically as never, past month, between 1 and 6 months, between 6 months and 1 year, and more than 1 year ago. We also asked two yes/no questions as to whether any overdose in the past year required emergency room treatment and whether they believed any past-year overdose was related to fentanyl.

Demographics collected were participant sex at birth (male or female), age in years, race/ethnicity in 5 categories (white, African American or black, Latino, other, multiracial) and the Chicago community location (northwest or west side) where they were recruited.

The survey included 6 questions that assessed current attitudes about DCS and fentanyl use. As examples: “I prefer fentanyl or drugs that have fentanyl in them; I am interested in being able to check my drugs for fentanyl before I use them; Most of the heroin on the street has fentanyl mixed in with it, there is not much point in testing heroin for fentanyl.” Participants responded to each question using a 5-point Likert-like scale that ranged from strongly disagree to strongly agree. We included an additional open-ended question to obtain participant responses as to how they would use DCS if available: “If you were able to check your drugs before using, what information about your drugs would be most important for you to have?”.

Of those who reported using drugs they believed contained fentanyl, we asked how they determined fentanyl was present. Participants could select one or more of 8 options such as “bought from the same source”, “used a test strip”, or “dealer said contained fentanyl”, etc. Each option was coded dichotomously as indicated/not indicated.

In the last part of the survey, we asked participants what drugs they believed were present in the sample provided for testing. They could select one or more drugs from a list of fifteen drugs centered on but not exclusive to opioids and which included fentanyl, heroin, oxycodone, codeine, methadone, diphenhydramine, MDMA, cocaine, benzodiazepines, and methamphetamine. An open-ended option was provided for participants to write in any unlisted drug they expected was in their sample.

### Analyses

We used R (version 4.1.3) statistical software [44] to conduct all analyses. We first calculated bivariate counts for each study measure disaggregated by overdose history category. Chi-square tests were used to assess statistical significance for categorical measures and one-way ANOVA’s were used for ratio level measures (e.g., age in years, number of past-year overdoses). Because there could be more than one drug expected in each sample as well as more than one drug detected through LC–MS testing, we generated an upset plot showing the expected drugs and drug combinations in the provided samples and as well as an upset plot for the drugs detected via LC–MS to provide a visual representation of expected versus actual drug combinations. The few open-ended questions were reviewed by the first author for summarizing as there was not enough qualitative data collected to warrant formal analysis.

## Results

### Demographics and overdose history

Sample demographics and overdose histories disaggregated by lifetime overdose category are presented in Table 1. A plurality of the sample was white (39.8%) followed by participants who identified as Latino (34.7%). A clear majority of participants (75.4%) indicated their gender at birth as male and the average age was just over 40 years (*M* = 40.9, SD = 9.7). Participants reported an average of 4.4 lifetime overdoses (SD = 4.8, range = 0–20) and an average of 1.1 (SD = 1.8, range = 0–10) past-year overdoses. Only 18 participants (15.3%) reported they have never overdosed. Of the 51 participants who overdosed in the past year, 60.8% said they required treatment in the emergency department, and almost all (97.9%) believed their overdose was due to fentanyl.

**Table 1:** Demographics and overdose history information

	Lifetime overdose category			Total (*N* = 118)	*p* value
	0–1 overdoses (*N* = 33)	2–5 overdoses (*N* = 54)	6+ overdoses (*N* = 31)		
*Chicago community location*					< .01
Northwest side	15 (45.5%)	31 (57.4%)	7 (22.6%)	53 (44.9%)	
West side	18 (54.5%)	23 (42.6%)	24 (77.4%)	65 (55.1%)	
*Race-ethnicity*					0.20
Black/African American	3 (9.1%)	10 (18.5%)	5 (16.1%)	18 (15.3%)	
White	18 (54.5%)	14 (25.9%)	15 (48.4%)	47 (39.8%)	
Latino	10 (30.3%)	23 (42.6%)	8 (25.8%)	41 (34.7%)	
Other	1 (3.0%)	1 (1.9%)	1 (3.2%)	3 (2.5%)	
Multi-racial/ethnic	1 (3.0%)	6 (11.1%)	2 (6.5%)	9 (7.6%)	
*Gender at birth*					0.07
Male	24 (72.7%)	37 (68.5%)	28 (90.3%)	89 (75.4%)	
Female	9 (27.3%)	17 (31.5%)	3 (9.7%)	29 (24.6%)	
*Age (years)*					0.43
Mean (SD)	42.37 (11.58)	41.35 (9.30)	39.26 (8.05)	41.09 (9.69)	
Median	40.2	41.5	40.5	40.5	
Min–max	23.00–67.70	26.60–60.20	23.30–56.90	23.00–67.70	
*Age category*					0.45
18–29	3 (9.1%)	4 (7.4%)	2 (6.5%)	9 (7.6%)	
30–39	12 (36.4%)	19 (35.2%)	11 (35.5%)	42 (35.6%)	
40–49	9 (27.3%)	17 (31.5%)	15 (48.4%)	41 (34.7%)	
50–59	6 (18.2%)	13 (24.1%)	3 (9.7%)	22 (18.6%)	
60 or older	3 (9.1%)	1 (1.9%)	0 (0.0%)	4 (3.4%)	
*# Lifetime overdoses*					< 0.001
Mean (SD)	0.45 (0.51)	3.04 (1.01)	10.87 (4.94)	4.37 (4.81)	
Median	0.0	3.0	10.0	3.0	
Min–max	0.00–1.00	2.00–5.00	6.00–20.00	0.00–20.00	
*# Past-year overdoses*					< 0.001
Mean (SD)	0.15 (0.36)	1.02 (1.21)	2.32 (2.84)	1.12 (1.85)	
Median	0.0	1.0	2.0	0.0	
Min–max	0.00–1.00	0.00–5.00	0.00–10.00	0.00–10.00	
*Most recent overdose*					< 0.001
Never overdosed	18 (54.5%)	0 (0.0%)	0 (0.0%)	18 (15.3%)	
Past month	1 (3.0%)	8 (14.8%)	7 (22.6%)	16 (13.6%)	
< 6 months	3 (9.1%)	15 (27.8%)	9 (29.0%)	27 (22.9%)	
Between 6 and 12 months	1 (3.0%)	6 (11.1%)	1 (3.2%)	8 (6.8%)	
More than 12 months	10 (30.3%)	25 (46.3%)	14 (45.2%)	49 (41.5%)	
*Any past-year OD need ER treatment?*					0.49
No	3 (60.0%)	10 (34.5%)	7 (41.2%)	20 (39.2%)	
Yes	2 (40.0%)	19 (65.5%)	10 (58.8%)	31 (60.8%)	
Missing/NA	28	25	14	67	
*Any past-year OD due to fentanyl?*					1.00
No	0 (0.0%)	1 (3.4%)	0 (0.0%)	1 (2.1%)	
Yes	5 (100.0%)	28 (96.6%)	14 (100.0%)	47 (97.9%)	
Missing/NA	28	25	17	70	

Significance tests for all nominal level variables were calculated Fisher's exact test and F tests based on one-way ANOVAs for all ratio level measures

Tests were run on the complete sample excluding 2 cases with no information for number of lifetime overdoses except for any past-year overdose needing ED treatment and past-year overdose due to fentanyl whereby participants with no past-year overdoses were also excluded

Statistical comparisons of these measures by lifetime overdose category did not yield significant differences for the demographic measures except for Chicago community location (*p* = 0.008). A much higher percentage of participants in the 6 + lifetime overdose category (77.4%) were recruited from the west side location compared with participants recruited from the northwest side office, who were most likely to be in the 2–5 lifetime overdose category (57.4%). Not surprisingly given that number of lifetime overdoses was used to construct the categorical indicator, the number of lifetime and past-year overdoses as well as most recent overdose category were all significantly different (*p* < 0.001), varying by lifetime overdose category. A higher proportion (22.6%) of those in the 6 + lifetime overdose category reported an overdose in the past month compared with those in the 2–5 overdose category (14.8%) and those in the 0–1 overdose category (3.0%) underscoring participants in this group continue to overdose frequently.

### Fentanyl preferences, drug checking attitudes, and fentanyl determination methods

Table 2 shows participant preference for, as well as concerns over, fentanyl and level of interest/receptiveness toward checking drugs prior to use, disaggregated by lifetime overdose category as well as overall. As none of the one-way ANOVAs assessing mean score differences by overdose category were statistically significant, our focus is on the overall sample statistics for each result.

**Table 2:** Drug checking services attitudes and fentanyl preference

	Lifetime overdose category			Total (*N* = 116)	*p* value
	0–1 overdoses (*N* = 32)	2–5 overdoses (*N* = 54)	6+ overdoses (*N* = 30)		
*Recently used drugs containing fentanyl*					0.48
Strongly disagree	0 (0.0%)	1 (1.9%)	1 (3.2%)	2 (1.8%)	
Disagree	2 (6.9%)	4 (7.4%)	0 (0.0%)	6 (5.3%)	
Neither agree/disagree	0 (0.0%)	1 (1.9%)	0 (0.0%)	1 (0.9%)	
Agree	17 (58.6%)	28 (51.9%)	15 (48.4%)	60 (52.6%)	
Strongly agree	10 (34.5%)	20 (37.0%)	15 (48.4%)	45 (39.5%)	
Median	3	3	3	3	
*Concerned drugs used contain fentanyl*					0.75
Strongly disagree	1 (3.1%)	2 (3.7%) 1	(3.3%)	4 (3.4%)	
Disagree	7 (21.9%)	12 (22.2%)	6 (20.0%)	25 (21.6%)	
Neither agree/disagree	1 (3.1%)	5 (9.3%)	3 (10.0%)	9 (7.8%)	
Agree	14 (43.8%)	24 (44.4%)	13 (43.3%)	51 (44.0%)	
Strongly agree	9 (28.1%)	11 (20.4%)	7 (23.3%)	27 (23.3%)	
Median	3	3	3	3	
*Prefer fentanyl/drugs with fentanyl*					0.31
Strongly disagree	6 (18.2%)	9 (17.0%)	10 (33.3%)	25 (21.6%)	
Disagree	14 (42.4%)	19 (35.8%)	7 (23.3%)	40 (34.5%)	
Neither agree/disagree	5 (15.2%)	0 (0.0%)	2 (6.7%)	7 (6.0%)	
Agree	7 (21.2%)	16 (30.2%)	7 (23.3%)	30 (25.9%)	
Strongly agree	1 (3.0%)	9 (17.0%)	4 (13.3%)	14 (12.1%)	
Median	3	3	3	1	
*Drugs with fentanyl look/taste different*					0.16
Strongly disagree	0 (0.0%)	2 (3.8%)	3 (10.3%)	5 (4.5%)	
Disagree	6 (20.0%)	8 (15.1%)	1 (3.4%)	15 (13.4%)	
Neither agree/disagree	5 (16.7%)	4 (7.5%)	1 (3.4%)	10 (8.9%)	
Agree	16 (53.3%)	26 (49.1%)	15 (51.7%)	57 (50.9%)	
Strongly agree	3 (10.0%)	13 (24.5%)	9 (31.0%)	25 (22.3%)	
Median	3	3	3	3	
*Interested in testing drugs for fentanyl*					0.32
Strongly disagree	0 (0.0%)	1 (1.9%)	0 (0.0%)	1 (0.9%)	
Disagree	3 (9.1%)	7 (13.5%)	1 (3.2%)	11 (9.5%)	
Neither agree/disagree	3 (9.1%)	2 (3.8%)	2 (6.5%)	7 (6.0%)	
Agree	19 (57.6%)	29 (55.8%)	17 (54.8%)	65 (56.0%)	
Strongly agree	8 (24.2%)	13 (25.0%)	11 (35.5%)	32 (27.6%)	
Median	3	3	3	3	
*Most heroin has fentanyl, no point testing*					0.77
Strongly disagree	3 (10.0%)	7 (13.5%)	4 (12.9%)	14 (12.4%)	
Disagree	16 (53.3%)	24 (46.2%)	13 (41.9%)	53 (46.9%)	
Neither agree/disagree	1 (3.3%)	4 (7.7%)	1 (3.2%)	6 (5.3%)	
Agree	7 (23.3%)	9 (17.3%)	6 (19.4%)	22 (19.5%)	
Strongly agree	3 (10.0%)	8 (15.4%)	7 (22.6%)	18 (15.9%)	
Median	1	1	1	1	
*Testing is too much trouble*					0.91
Strongly disagree	7 (21.2%)	10 (19.2%)	9 (30.0%)	26 (22.6%)	
Disagree	17 (51.5%)	24 (46.2%)	8 (26.7%)	49 (42.6%)	
Neither agree/disagree	1 (3.0%)	6 (11.5%)	4 (13.3%)	11 (9.6%)	
Agree	6 (18.2%)	8 (15.4%)	8 (26.7%)	22 (19.1%)	
Strongly agree	2 (6.1%)	4 (7.7%)	1 (3.3%)	7 (6.1%)	
Median	1	1	1	1	

Total *N* (116) excludes 2 cases with missing data on lifetime overdose category and an additional 2 cases with missing data on the attitudes scale questions. All significance tests were run using the Kruskal–Wallis equality-of-populations rank test

The results for fentanyl preference were mixed. A large majority (92.1%) of participants, agreed/strongly agreed they had recently used fentanyl or drugs containing fentanyl and over two-thirds (67.3%), said they were concerned their street drugs contained fentanyl. Although most participants believed their street drugs (sold mainly as heroin) contained fentanyl, a majority (56.1%) disagreed/strongly disagreed they preferred fentanyl or drugs containing fentanyl. On the other hand, a sizeable minority, over one-third (38.0%), indicated they *do* prefer fentanyl or drug mixtures containing it.

Attitudes toward drug checking indicated a general but not uniform receptiveness. A large proportion of participants (83.6%) said they agreed/strongly agreed they would be interested in testing drugs for fentanyl. More than half (59.3%) disagreed/strongly disagreed with the statement “as most heroin has fentanyl, there is no point in testing” and 65.2% also disagreed/strongly disagreed that “testing drugs before use was too much trouble”. Again, for both questions a sizeable minority endorsed the response options indicating a lack of interest in drug checking as a 25.2% agreed/strongly agreed that “drug testing was too much trouble” and over one-third (35.4%) agreed “there was no point in testing”.

We next coded the open-ended responses from the subset of 31 participants from whom we obtained qualitative data on what specific information they would want to get from drug checking; their responses were classified into four categories. Ordered from the most to least frequently mentioned, participants said they would want to use drug checking to know: the potency or concentration of drugs including but not exclusive to fentanyl (*N* = 14, 45.1%); what specific drugs were in the overall mixture (*N* = 11, 35.5%); whether harmful, unexpected cutting agents had been used (*N* = 9, 29.0%); and whether fentanyl was present (*N* = 8, 25.8%).

**Fig. 1:**
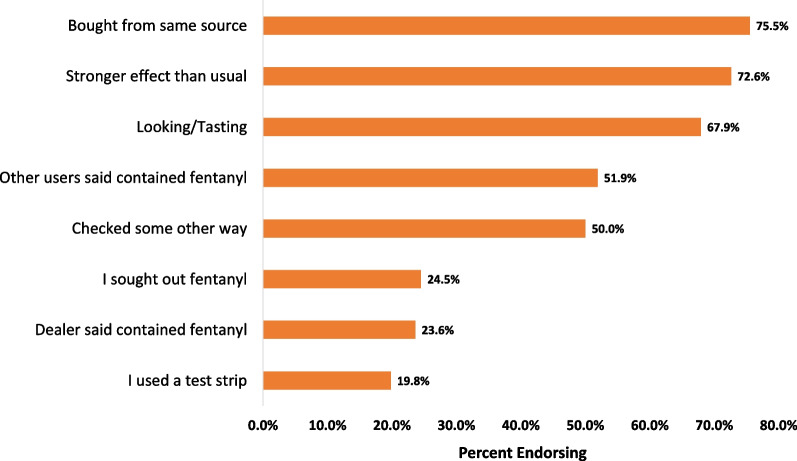
Preferred methods for determining the presence of fentanyl in street drugs

We asked participants whether they believed they had taken any drugs in the past month that contained fentanyl. Just over 88% (*N* = 106) of the 118 participants said they had, with 6 (5.0%) additional participants indicating they were unsure and 6 (5.0%) indicating they had not. When participants were asked what method(s) they relied upon to determine the presence of fentanyl in their drugs (Fig. 1), the most common response (75.5%) was buying from the same source followed by noticing their drugs had a stronger effect than usual (72.6%). Despite the previously cited research showing the appearance of drugs is not a reliable way to detect fentanyl [32], judging by the appearance or taste of their drugs (67/9%) was the third most common determination method. Using a test strip was the least frequently reported method for fentanyl determination (19.8%). However, we note that test strips had not been widely available at the SSPs for most of the study period, possibly influencing how often this option was endorsed.

Exactly half of participants responding to this question said they used some other way to determine their sample contained fentanyl. Reviewing the open-ended responses provided to explain this selection, about half indicated they knew based on either the effects of taking the drug (e.g., experienced a black-out, shorter duration of effects, etc.) or based on a urinalysis result when they were tested during a hospitalization or at a methadone treatment clinic. In other words, these participants were not pre-determining their drugs contained fentanyl before using, they only made that determination after-the-fact.

The next most common explanation was that the color of the drug was different, sometimes revealed after adding water and beginning to heat the drug (i.e., “cooking”) prior to injection. Among those who provided this explanation, there were numerous mentions of the drug turning purple or pinkish during preparation.

Although we could include these responses with identification of fentanyl through the drug’s appearance, they could also reflect two relatively recent and distinct phenomena with respect to the composition of illegally sold opioids. The first is the novel psychoactive substance, brorphine. Described as “purple fentanyl”, brorphine has been synthesized specifically for the illegal drug market and used to fully supplant or included in mixture with fentanyl [45–48]. Brorphine was first widely detected in street drug samples in midwestern states such as Michigan and Illinois, but has since become more widespread in the USA [45]. It is as potent as fentanyl and has been increasingly responsible for opioid-related overdoses and fatalities in the past few years, underscoring the potentially fatal hazard of trying to assess the composition of a street drug sample by its appearance.

### Expected versus detected drug mixtures

A total of 109 participants had both survey results for what drugs they expected to be in their provided sample and LC–MS results when the provided sample was thought to contain an opioid. On average, participants expected their samples contained a mixture of 3.01 drugs (SD = 1.42, range = 1–8). The LC–MS results detected fewer drugs present, however (mean = 2.31, SD = 0.68, range = 1–4), a statistically significant difference (*t* = 4.39, *df* = 152.91, *p* < 0.001).

**Fig. 2:**
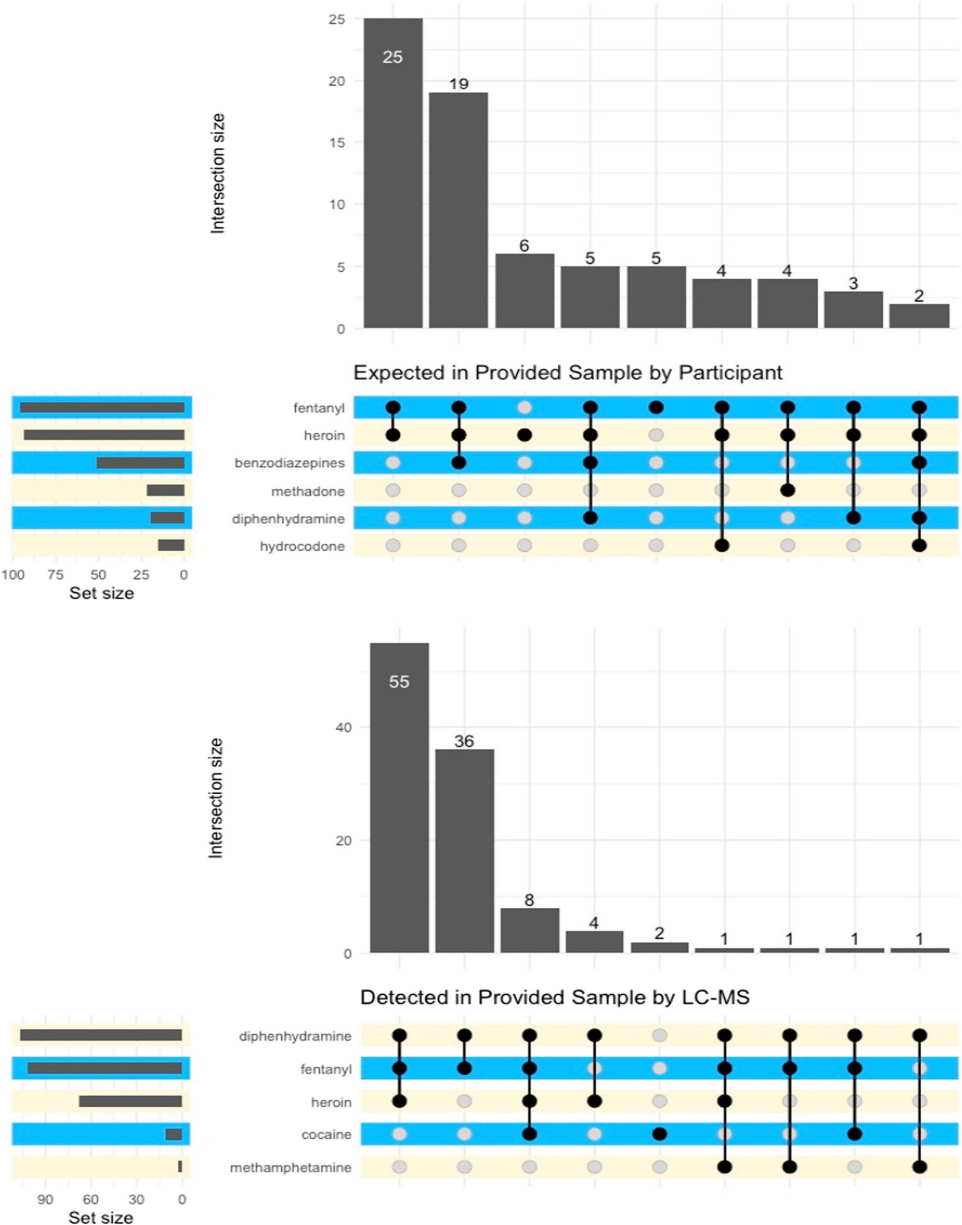
Expected and detected drug mixtures in provided samples. *Note*: Results based on the 109 participants who provided both survey data on expected drugs in their sample and a street drug sample that could be tested via LC–MS. The samples assessed here were restricted to those identified by participants as containing opioids. *Intersection size* reflects the total number of times a given combination was expected or detected, whereas *set size* reflects the total number of times a drug was expected or detected, regardless of in what combination with other drugs

Figure 2 shows that whereas participants expected a wide variety of drugs to be present in the provided samples, their perceptions were often inaccurate based on the expected combinations. Most striking was the extent to which they underestimated how frequently diphenhydramine was contained in their drugs (sensitivity = 0.17) and overestimated the presence of benzodiazepines (specificity = 0.51). The most frequently expected combinations were fentanyl and heroin (*N* = 25. 22.9%) or fentanyl, heroin, and benzodiazepines (*N* = 19, 17.4%).

The LC–MS test results show that most of the street samples included a mixture of drugs (not including cutting agents) but that it was a much more limited set of drugs than expected by the participants. The most common detected mixture was diphenhydramine, fentanyl, and heroin (*N* = 55, 38.9%). A high percentage (*N* = 41, 36.6%) of the samples contained no heroin at all but were instead a mixture of diphenhydramine and fentanyl. Only 1 sample contained heroin exclusively. As we focused this study on opioid users, only 3 of the samples provided contained cocaine, 2 or which were cocaine only and the other 2 containing fentanyl and heroin, as well as levamisole, consistent with the previously cited report of this cutting agent showing up with increasing frequency in cocaine.

## Discussion

Although most study participants correctly expected their opioid street drugs contained fentanyl, a majority said they still saw benefit of having DCS available and that drug checking was not “too much trouble”. Hence, interest in DCS has not been appreciably eroded among opioid street drug users despite the current predominance of fentanyl in the illegal opioid supply [15]. This finding is also consistent with similar research conducted in Canada within the last few years [26].

Counter to prior studies, we did not find that interest in DCS varied by overdose history. We had expected persons with more prior overdoses would express the most interest in DCS, but this was not the case; participants with less frequent or no prior overdoses were equally supportive of drug checking to test their drugs. Here, it could be important to note that drug checking not only can prevent opioid-related overdoses and fatalities, it can also help identify cutting and potentiating agents that deleteriously affect health in other ways. Appreciation of the broader health benefits of drug checking could partially underlie why we found no variation in interest by overdose history.

Despite the continued interest in DCS, we also found that many participants continue to place their trust in inaccurate methods to determine what drugs are present in their sample; these included using a “trusted” dealer, assessing the appearance of the drug, or even assessing after-the-fact effects of using the drug [32]. One unfortunate consequence of using such informal assessment methods was the continuing high rates of opioid-related overdoses among study participants. For the majority of our participants then, making valid and reliable DCS available, even in a time when fentanyl appears to be much more common than heroin or other drugs sold as opioids, could provide them with useful and potentially life-saving information about the street drugs they are about to ingest.

As noted, cutting and potentiating agents that can have significant adverse health consequences such as levamisole and diphenhydramine are commonly present in street drug samples. Based on the LC–MS testing done, we found most of the samples provided contained multiple drugs and that participants were especially poor at assessing the presence of the full range of constituent drugs in their samples. For this reason, we strongly believe that DCS would be maximally beneficial if it provided information about as many of the constituent drugs within a sample as possible and not just information about a single drug.

Similarly, we also believe based on participant responses to an open-ended qualitative question at the end of the survey on how drug checking could be most helpful and our own observations, that opioid (and other) street drug users would especially value having quantitative information on the concentration of a drug or drugs present and not just qualitative identification of the presence/absence of the drug or drugs present. As most of our study participants expected fentanyl to be present in their street drugs, the questions they asked of the RA who collected the drug samples for testing were more about the concentration/dosage; they often said they wanted to know how much fentanyl was in the sample they provided.

Providing information on multiple drugs and quantifying concentrations in street drug samples, while perhaps optimal, present their own challenges. For one, more comprehensive and sensitive testing methods are expensive, require varying levels of technical expertise, and are not generally available for street drug users to conduct their own testing. Additionally, some advanced testing methods require destruction of at least a part of the sample which, as noted in the introduction, can be a barrier for some users. With the current status of drug testing technology, to get comprehensive and detailed information on the constituent drugs in provided samples, users have to have their drugs tested on-site at a well-equipped community services center with trained staff or at a dedicated drug testing laboratory.

The delay and inconvenience in getting on-site test results might dissuade more than a few street drug users from using advanced DCS through a clinic or drug testing laboratory. More convenient take-home methods such as fentanyl or benzodiazepine test strips, while inexpensive and highly sensitive to detecting the presence or absence of the drug for which they were designed, provide limited, qualitative information about street drug samples. Moreover, our study data suggest that street opioid users not only expect their drugs to contain fentanyl but that some seek out street drugs that contain fentanyl because they prefer this drug. Consequently, for many opioid street drug users testing their drugs for the presence or absence of fentanyl might not provide especially useful information.

Fentanyl test strips could possibly have the most benefit when used to test street drugs *not* sold as opioids (e.g., cocaine, MDMA, methamphetamine). Lacking tolerance for opioids, users of (expected) non-opioid street drugs can experience overdoses and fatalities when even a very small amount of a potent opioid such as fentanyl or analog is present in their drug. Recent publications in the scientific literature and popular press indicate adulteration of non-opioids with fentanyl is occurring with more frequency, often having fatal consequences [1, 49–51].

As drug testing methods evolve, however, more sensitive and comprehensive testing methods such as portable gas chromatograph-mass spectrometry and Fourier-transform infrared spectroscopy (FTIR) that can be provided as point-of-care testing are being evaluated and could soon come into wider use [16, 30, 52, 53]. These testing methods represent what we assess as a reasonable compromise between more advanced but expensive and technologically complex testing methods and accessible, easy-to-use but less informative methods available for take-home use. Some programs, notably those established in Canada, have incorporated multiple means of implementing DCS that offer options to users desiring their drugs be tested. The options range from the one used in our study as the gold-standard, LC–MS, to mobile FTIR devices, to providing program users with single-drug test strips. If affordable (and politically/legally feasible [16, 54]), offering a range of DCS technologies seems the best possible model at present [24, 55]. Regardless of the technologies available however, the rapidly changing street drug market and the seemingly constant evolution and availability of new analog synthetic opioids will present continued challenges to any testing technology used to provide DCS to street drug users [17]. Drug checking technologies will have to also be capable of rapidly evolving to detect newly synthesized drugs as they are added to the illegal drug supply.

## Limitations

This study collected data from Chicago street drug users recruited because they indicated they had recently purchased illegally manufactured opioids and were seeking services at an SSP. The attitudes toward DCS might not generalize to all settings and street drug using populations within and outside the USA. We also note that data were collected at a time when fentanyl and benzodiazepine test strips were not widely available through the outreach program where the study was conducted. This too could have influenced participant attitudes toward and acceptance of DCS. Although we considered running formal statistics to estimate the sensitivity, specificity, and overall concordance between user’s estimates of what their street drug samples contained and the laboratory-test results, the discrepancies were so large that these statistics were relatively meaningless. Aside from believing their street drugs contained fentanyl, which was fairly accurate, most users were not aware of what other drugs and cutting agents were also present. As our focus was on persons using opioids, we collected only a few street drug samples that users believed to be primarily composed of other drugs such as cocaine. Given the noted issues with fentanyl now being used in many non-opioid drugs sold on the street in powder and pill form, assessing DCS for a range of street drugs seems an especially important area for continued research.

## Conclusions

Despite the predominance of fentanyl in illegally manufactured opioid street drugs, we found users continued to express interest in DCS to help them assess the contents of the drugs they were about to ingest not only for fentanyl and related analogs but also for the presence of other drugs and cutting agents. Accordingly, we found most of the street drug samples tested contain multiple drugs besides fentanyl that could have significant health effects. The street drug users we interviewed expressed interest in drug checking technologies that provide information on drug concentrations and identification of all the constituent drugs in their samples. Having advanced drug checking technologies that can provide this information widely available at point-of-care street outreach and SSPs continues to present significant technical, practical, and economic challenges but is worth pursuing considering the large, ongoing, and adverse public health effects of the opioid epidemic and associated fatalities.

## Data Availability

The data set analyzed for the current study are available from the corresponding author on reasonable request.

## Abbreviations

DCS: Drug checking services
DF: Degrees of freedom
FTIR: Fourier-transform infrared spectroscopy
LC–MS: Liquid chromatography–mass spectrometry
MDMA: 34-Methylenedioxy-methamphetamine (i.e., “Ecstasy”)
MOUD: Medication for opioid use disorder
OD: Overdose/overdoses
OEND: Opioid education and naloxone distribution
PDMP: Prescription drug monitoring programs
SD: Standard deviation
SSP: Syringe service programs
